# Association between nutrition-focused evidence-based nursing and post-operative nutritional status in patients undergoing colorectal cancer surgery

**DOI:** 10.3389/fnut.2026.1784385

**Published:** 2026-08-07

**Authors:** Li-Na Song, Yi-Fan Gu, Nan Jin, Pei-Ming Sun, Shu-Zhen Wei, Jie Zhang

**Affiliations:** 1 Department of General Surgery, The Ninth Medical Center of PLA General Hospital, Beijing, China; 2 Department of Gastroenterology, The Ninth Medical Center of PLA General Hospital, Beijing, China; 3 Department of Pediatrics, The Ninth Medical Center of PLA General Hospital, Beijing, China; 4 Department of Nursing, The Ninth Medical Center of PLA General Hospital, Beijing, China; 5 School of Nursing, Southern Medical University, Guangzhou, Guangdong, China

**Keywords:** colorectal cancer surgery, enteral nutrition, nutritional risk screening 2002, nutrition-focused evidence-based nursing, oral nutritional supplements, parenteral nutrition, post-operative nutritional risk

## Abstract

**Background:**

Patients undergoing colorectal cancer surgery are at risk of postoperative nutritional deterioration due to perioperative metabolic stress, inflammation, and transient gastrointestinal dysfunction. Evidence-based, nursing-led nutritional pathways may improve perioperative nutritional surveillance and timely nutrition support.

**Methods:**

This retrospective study included 214 adults who underwent elective curative-intent colorectal cancer resection between January 2023 and December 2024. Patients receiving routine perioperative nursing care in 2023 were assigned to the control group (*n* = 106), whereas those receiving the nutrition-focused evidence-based nursing protocol in 2024 were assigned to the observation group (*n* = 108). The primary outcomes were change in Nutritional Risk Screening 2002 (NRS-2002) score and postoperative nutritional risk, defined as NRS-2002 ≥ 3 at postoperative day 7 or immediately before discharge.

**Results:**

Baseline characteristics were comparable between groups. The observation group had lower postoperative NRS-2002 scores and smaller ΔNRS-2002 than the control group (both *P* < 0.001). Postoperative nutritional risk was also lower in the observation group (44.4% vs. 67.0%, *P* = 0.001). After multivariable adjustment, the observation group remained associated with lower odds of postoperative nutritional risk (adjusted odds ratio = 0.17, 95% confidence interval: 0.08–0.36; *P* < 0.001). Secondary findings showed less weight loss, smaller declines in albumin and prealbumin, lower discharge C-reactive protein, earlier feeding recovery, shorter postoperative hospital stay, fewer overall complications, and fewer nutrition-support–related safety events.

**Conclusions:**

A nutrition-focused evidence-based nursing protocol was associated with more favorable postoperative nutritional risk status assessed by NRS-2002, as well as selected short-term recovery- and safety-related outcomes after colorectal cancer surgery. These findings should be interpreted as associative and require confirmation in prospective studies.

## 1 Introduction

Colorectal cancer surgery is frequently accompanied by substantial metabolic stress, perioperative inflammation, and transient gastrointestinal dysfunction, all of which may precipitate clinically relevant deterioration in nutritional status (1, 2). Cancer-related malnutrition is multifactorial and involves reduced intake, catabolic signaling, micronutrient imbalance, and impaired antioxidant capacity, which collectively may increase susceptibility to postoperative infectious and anastomotic complications and delay functional recovery (2, 3). Systematic perioperative nutritional risk screening is therefore integral to contemporary colorectal perioperative care. The Nutritional Risk Screening 2002 (NRS-2002) is a widely implemented hospital-based tool that integrates nutritional impairment and disease severity and supports timely escalation to targeted nutritional interventions (4). Recent clinical studies continue to demonstrate that higher perioperative nutritional risk, as assessed by screening instruments including NRS-2002 and related frameworks, correlates with adverse postoperative outcomes and resource utilization, reinforcing the need for standardized, protocol-driven nutritional surveillance and intervention (5–8). However, implementation of structured nutritional screening, assessment, and early postoperative feeding remains heterogeneous across institutions and clinical pathways, indicating persistent practice gaps that may be amenable to quality-improvement strategies (9–11).

Multiple nutrition-focused measures have been evaluated to mitigate postoperative nutritional decline after gastrointestinal oncologic surgery, including early oral intake, oral nutritional supplements (ONS), enteral nutrition (EN), parenteral nutrition (PN) when indicated, and immunonutrition strategies (12). Evidence syntheses and clinical studies published in recent years support that earlier feeding and structured nutrition protocols can improve nutritional trajectories and selected recovery milestones, although the magnitude of benefit varies across study designs, patient risk profiles, and intervention fidelity (13–15). Importantly, perioperative nutritional interventions are operationally complex, requiring repeated screening, goal setting (energy/protein targets), monitoring of intake and tolerance, prevention and management of feeding-related adverse events, and coordination across disciplines.

Within this context, nursing teams are positioned to translate evidence into routine practice by embedding standardized screening, risk-stratified nutrition plans, dynamic monitoring, and escalation algorithms into perioperative workflows. A nutrition-focused evidence-based nursing model may therefore provide a pragmatic implementation framework to reduce postoperative nutritional risk and support recovery after colorectal cancer surgery, while enabling consistent delivery of nutrition care in real-world ward settings.

## 2 Methods

### 2.1 Study design

This retrospective study consecutively enrolled patients who underwent surgery for colorectal cancer at our institution between January 2023 and December 2024. Patients treated from January 2023 to December 2023 received routine perioperative nursing care and were assigned to the control group (*n* = 106), whereas patients treated from January 2024 to December 2024 received a nutrition-focused evidence-based nursing protocol and were allocated to the observation group (*n* = 108). The study's design, objectives, and protocols were aligned with the STROBE (Strengthening the Reporting of Observational Studies in Epidemiology) guidelines (16). Eligible participants were adults (≥18 years) with pathologically confirmed colorectal cancer who underwent elective curative-intent resection, had complete perioperative clinical data and nutritional assessments available, and were able to cooperate with nutritional screening, education, and follow-up. Exclusion criteria comprised emergency surgery; concurrent or previous malignancy within the past 5 years; severe hepatic, renal, or cardiac dysfunction; inflammatory bowel disease; pre-existing conditions markedly affecting nutrition assessment (e.g., decompensated liver cirrhosis, nephrotic syndrome); preoperative parenteral nutrition or enteral tube feeding initiated before admission; pregnancy or lactation; perioperative admission to the intensive care unit with mechanical ventilation; and missing key outcome data. During the study period, the institutional perioperative framework for colorectal surgery, including ERAS-based perioperative care, anesthesia routines, postoperative monitoring, and discharge criteria, remained unchanged. All colorectal procedures in both periods were performed within the same institutional colorectal surgery department and surgical service. The study was approved by the hospital's ethics committee and conducted in accordance with relevant guidelines and the Declaration of Helsinki. Informed consent was obtained from all participants. All personal data were anonymized prior to analysis to ensure confidentiality and protect participant privacy.

### 2.2 Perioperative nursing protocols

Control group (routine perioperative nursing care). Patients in the control group received standard perioperative nursing care according to institutional routines. (1) Upon admission, nurses conducted routine preoperative assessment (medical history review, vital signs monitoring, perioperative safety checks) and delivered general perioperative education, including fasting requirements, bowel preparation instructions, basic respiratory training, and guidance on early mobilization and pain reporting. (2) Postoperatively, diet advancement (e.g., water to liquid, semi-liquid, and soft/regular diet) was implemented primarily based on surgeon orders and patient tolerance; nurses monitored gastrointestinal symptoms (nausea/vomiting, abdominal distension, bowel sounds, passage of flatus/defecation) and provided routine supportive care, including assistance with analgesia adherence, wound/stoma care when applicable, and prevention of common postoperative complications. (3) Nutritional support beyond usual meals (e.g., oral nutritional supplements, enteral feeding, or parenteral nutrition) was provided only when prescribed by the treating team or when clinically indicated, without a standardized nursing-led nutrition risk screening, individualized energy/protein target setting, or protocolized intake-adequacy evaluation. (4) At discharge, patients received conventional discharge counseling covering general dietary advice, medication use, activity recommendations, and follow-up arrangements per routine practice, without structured post-discharge nutrition reassessment by nursing.

Observation group (nutrition-focused evidence-based nursing). Patients in the observation group received a structured, nutrition-focused evidence-based nursing protocol integrated into the perioperative pathway. (1) Within 24 h of admission, nurses performed standardized nutrition risk screening using NRS-2002 and documented baseline nutrition-related variables, including anthropometrics, recent unintentional weight loss, and current intake reduction, followed by individualized risk stratification to guide intervention intensity. (2) Before surgery, an individualized nursing nutrition plan was developed based on screening results and clinical status, including goal-oriented intake management, patient/family education on protein-focused dietary strategies, symptom-oriented eating advice, and initiation of ONS when indicated; patients considered at high nutritional risk according to institutional practice, such as those with NRS-2002 ≥ 3, marked recent unintentional weight loss, substantial preoperative intake reduction, or anticipated inability to meet nutritional targets through oral intake alone, were referred for dietitian/physician co-management. According to institutional practice, daily energy and protein targets were generally set at 25–30 kcal/kg/day and 1.2–1.5 g/kg/day, respectively. When routine oral intake was judged insufficient to meet the planned targets, high-protein ONS were prescribed, typically 200–250 mL per serving, 2–3 times daily, with dose adjustment based on the estimated intake gap and tolerance. (3) After surgery, early oral feeding was promoted when clinically permissible, with tolerance-guided diet progression from water to liquid, semi-liquid, and soft/regular diet, and systematic monitoring of intake adequacy against the planned targets. Intake adequacy was assessed daily; when total oral intake remained < 60% of target requirements for 48–72 h, a step-up strategy was activated in collaboration with the medical team. EN was considered when gastrointestinal function was adequate but oral intake remained insufficient, whereas PN was considered when EN was contraindicated, poorly tolerated, or when combined oral/enteral intake still remained < 60% of targets after 72 h. The structured reassessment and escalation algorithm used to guide daily intake monitoring, nutritional insufficiency assessment, and step-up nutrition support in the observation group is provided in Supplementary Material 1. (4) Throughout hospitalization, nurses conducted scheduled reassessments of nutrition risk and nutrition-related symptoms, including nausea/vomiting, pain, and bowel function, implemented evidence-based symptom management to facilitate intake, and documented protocol implementation in routine nursing records using standardized forms where available. (5) Prior to discharge, nurses provided a written, individualized nutrition plan, including diet progression, high-protein strategies, supplement use when needed, and stoma-specific dietary guidance where applicable, and conducted protocolized post-discharge follow-up (e.g., telephone-based reassessment) to evaluate intake, weight change, and gastrointestinal tolerance, with timely referral for nutrition clinic review when deterioration or persistent inadequacy was identified.

### 2.3 Variables and data collection

Clinical variables were retrospectively extracted from the electronic medical record and nursing information systems using a standardized case report form. (1) Baseline demographic and lifestyle variables included age (years), sex (male/female), height and weight measured at admission, and body mass index (BMI, kg/m^2^) calculated as weight/height^2^; smoking and alcohol use were recorded when available; baseline nutritional risk was assessed using the NRS-2002 and documented as the total score. (2) Disease- and treatment-related variables comprised tumor location (colon/rectum and, if available, segment), tumor stage based on pathological TNM reports, surgical approach (laparoscopic/open), procedure type (right/left hemicolectomy, sigmoid colectomy, anterior resection, abdominoperineal resection), stoma status (none/temporary/permanent), anesthesia type, operative duration (min), estimated blood loss (mL), perioperative transfusion (yes/no), receipt of preoperative chemotherapy and/or radiotherapy (yes/no), comorbidities (diabetes mellitus, anemia, chronic kidney disease), and ASA physical status classification. (3) Perioperative nursing and nutrition support variables included the use of ONS, EN, and PN (yes/no), with initiation time and duration recorded when available, as well as feeding route; nutrition delivery adequacy relative to prespecified targets was captured when documented in routine records. (4) Laboratory nutrition-inflammation indices included serum albumin, prealbumin, total protein, hemoglobin, and C-reactive protein (CRP), recognizing that serum protein markers may reflect both nutritional status and postoperative inflammatory burden. Preoperative baseline values were defined as the last available measurements obtained before surgery. For discharge-related analyses, the pre-discharge value was defined as the last postoperative laboratory measurement obtained within 24 h before hospital discharge; if more than one measurement was available within this window, the value closest to discharge was selected. Accordingly, “CRP at discharge” refers to the last postoperative CRP value obtained within 24 h before discharge, and ΔCRP was calculated as the discharge CRP minus the preoperative baseline CRP. (5) The weight of patients was measured upon admission and at discharge. The discharge weight was recorded as the last available measurement obtained within 24 h prior to hospital discharge. (6)Data quality control was ensured by double data extraction and consistency checks; units were standardized, and implausible values were verified against source records and treated as missing if unverifiable.

### 2.4 Outcomes

Outcomes were predefined and obtained from the electronic medical record, nursing documentation, and laboratory information system. (1) The primary postoperative NRS-2002 assessment was pre-specified as the POD7 assessment when available; for patients discharged before POD7 or without a POD7 record, the last available assessment before discharge was used, calculated as ΔNRS-2002 = NRS-2002_post – NRS-2002_pre. In this study, postoperative NRS-2002 was used as a pragmatic, clinically implemented indicator of postoperative nutritional risk status, derived according to routine institutional practice using the standard NRS-2002 framework, including the disease severity component, and no modified recalculation excluding this component was performed. A reduction in score was interpreted as improvement. The proportion of patients with nutritional risk (commonly defined as NRS-2002 ≥ 3) at the postoperative assessment was also reported. (2) Secondary outcomes included nutrition-related endpoints (nutrition adequacy rate during hospitalization when intake/target records were available, incidence of postoperative nutritional risk/malnutrition based on NRS-2002 categorization, and use and duration of oral nutritional supplements, enteral nutrition, and parenteral nutrition) and clinical endpoints (time to first flatus and first defecation, time to diet resumption/tolerance of oral intake, length of postoperative hospital stay, 30-day readmission, and postoperative complications such as surgical site infection, anastomotic leakage, postoperative ileus/intestinal obstruction, and pulmonary infection). (3) Safety endpoints comprised nutrition support–related adverse events documented in routine care, including nausea/vomiting or diarrhea requiring escalation, suspected aspiration events, catheter-related complications, hyperglycemia requiring intervention, and clinically significant electrolyte disturbances. (4) Outcome ascertainment was performed by reviewers not involved in nursing delivery, and complications were adjudicated using prespecified definitions supported by source documentation (progress notes, imaging, laboratory findings, and treatment initiation records). A supplementary discharge-time indicator of nutrition-related deterioration was also evaluated and pragmatically defined, according to institution-specific clinical practice, as serum albumin < 35 g/L and/or ≥5% weight loss from baseline.

### 2.5 Statistical analysis

Statistical analyses were performed using IBM SPSS Statistics version 28.0 (IBM Corp., Armonk, NY, USA). Continuous variables were tested for normality using the Shapiro–Wilk test and are presented as mean ± standard deviation or median (interquartile range), as appropriate; between-group comparisons were performed using the independent-samples *t* test or Mann–Whitney *U* test. Categorical variables are presented as number (percentage) and were compared using the chi-square test or Fisher's exact test, as appropriate. The two prespecified primary outcomes were ΔNRS-2002 (postoperative minus preoperative) and postoperative nutritional risk, defined as NRS-2002 ≥ 3 at postoperative day 7 or immediately before discharge. Multivariable linear regression was used for ΔNRS-2002, and multivariable binary logistic regression was used for postoperative nutritional risk. Prespecified covariates included age, sex, body mass index, smoking status, alcohol use, preoperative NRS-2002, tumor site, TNM stage, surgical approach, stoma status, operative duration, estimated blood loss, perioperative transfusion, preoperative chemo/radiotherapy, ASA class, diabetes mellitus, anemia, and chronic kidney disease. Results are reported as β coefficients or odds ratios with 95% confidence intervals. Subgroup analyses were conducted for tumor site, surgical approach, stoma status, and preoperative nutritional risk by including group-by-subgroup interaction terms. Sensitivity analyses were performed using alternative covariate sets and an alternative definition of postoperative nutritional risk (NRS-2002 ≥ 4). Model performance for logistic regression was assessed using the C-statistic, calibration intercept, calibration slope, calibration plot, Hosmer–Lemeshow goodness-of-fit test, and Brier score. Linear regression diagnostics included residual-vs.-fitted plots, normal Q–Q plots, Cook's distance, and adjusted R^2^. Multicollinearity was assessed using variance inflation factor and tolerance, with VIF < 5 considered acceptable. A *post-hoc* multivariable logistic regression analysis was additionally performed for any postoperative complication. All tests were two-sided, and P < 0.05 was considered statistically significant. Analyses of the two primary outcomes were considered confirmatory, whereas secondary, safety, subgroup, and sensitivity analyses were considered exploratory/supportive; no formal multiplicity adjustment was applied. Primary outcomes were analyzed using a complete-case approach, and secondary and safety outcomes using an available-case approach without imputation.

## 3 Results

### 3.1 Study flow and patient enrollment

**Figure 1:**
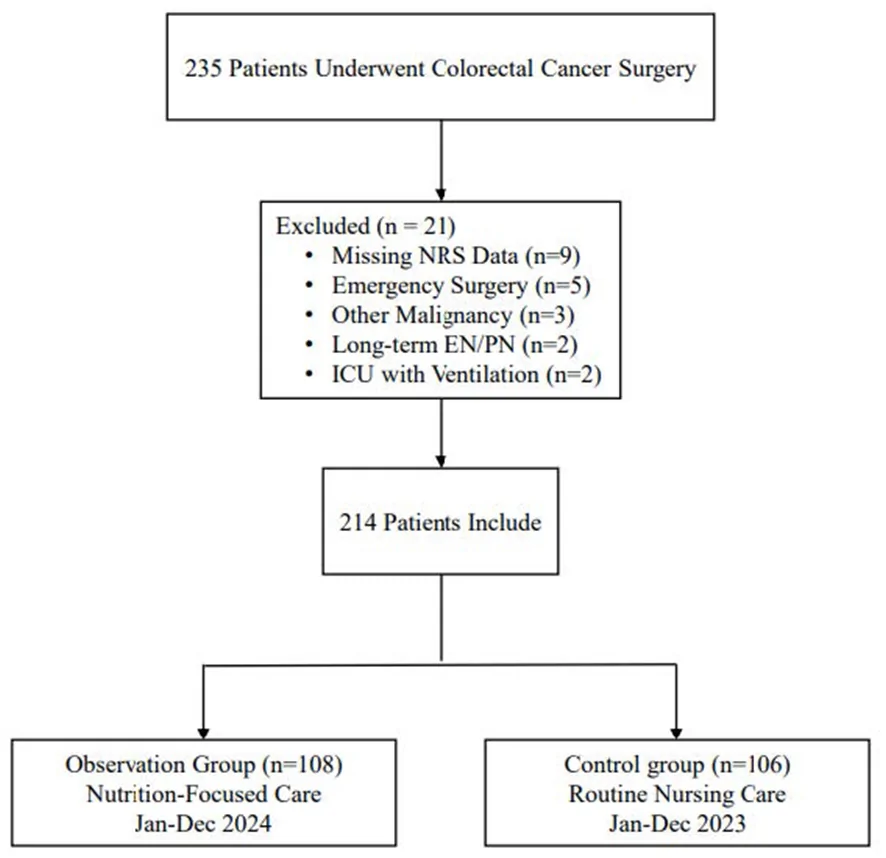
Patient enrollment and study flow.

Among 235 consecutive patients screened for eligibility, 214 were included in the final analysis after exclusions, including 106 in the control group and 108 in the observation group (Figure 1). The primary outcome analysis was conducted using a complete-case approach; all included patients had available NRS-2002 assessments at baseline and at the postoperative assessment time point (postoperative day 7 when available or the last assessment before discharge), and therefore no missing data were present for the primary endpoint. For selected secondary outcomes with incomplete availability (e.g., certain follow-up laboratory indices or 30-day outcomes), analyses were performed using the available-case dataset, and the corresponding denominators were reported where applicable. The distribution of postoperative NRS-2002 assessment days was comparable between the two groups and is provided in Supplementary Table S2. The median assessment day was POD7 in both groups (IQR: 7–8), and similar proportions of patients were assessed exactly on POD7 in the control and observation groups (56.6% vs. 56.5%). Fifteen patients (14.2%) in the control group and 18 patients (16.7%) in the observation group were assessed before POD7 because of early discharge.

### 3.2 Baseline characteristics and between-group comparability

Baseline demographic, anthropometric, lifestyle, and clinicopathologic characteristics were broadly similar between the control group and the observation group. No statistically significant between-group differences were observed in age, sex, body weight, BMI, smoking status, or alcohol use (all *P* > 0.05). Preoperative nutritional status was also comparable, with no significant differences in baseline NRS-2002 score or in the proportion of patients with NRS-2002 ≥ 3 (both *P* > 0.05). In addition, disease- and treatment-related variables, including tumor location, pathologic TNM stage, surgical approach, procedure type, stoma status, anesthesia type, operative duration, estimated blood loss, perioperative transfusion, preoperative chemo/radiotherapy, ASA physical status, and major comorbidities, did not differ significantly between the two groups (all *P* > 0.05) (Table 1).

**Table 1:** Baseline demographic, lifestyle, and clinicopathologic characteristics of patients undergoing colorectal cancer surgery, by study group.

Variable	Control (*n* = 106)	Observation (*n* = 108)	Test statistic	*P* value
Age, years	63.15 ± 9.40	61.98 ± 9.83	*t* = 0.89	0.374
BMI, kg/m^2^	23.81 ± 3.11	24.03 ± 2.90	*t* = −0.53	0.596
Weight, kg	65.18 ± 11.23	65.03 ± 10.07	*t* = 0.10	0.920
Male sex, *n* (%)	60 (56.6)	54 (50.0)	χ^2^ = 0.69	0.406
Smoking status (Never/Former/Current), *n* (%)	69 (65.1)/13 (12.3)/24 (22.6)	63 (58.3)/18 (16.7)/27 (25.0)	χ^2^ = 1.24	0.539
Alcohol use (Never/Former/Current), *n* (%)	71 (67.0)/11 (10.4)/24 (22.6)	78 (72.2)/7 (6.5)/23 (21.3)	χ^2^ = 1.22	0.543
Baseline NRS-2002 score, median (IQR)	2 (1–3)	2 (1–3)	Z = −0.96	0.325
NRS-2002 ≥ 3, *n* (%)	44 (41.5)	53 (49.1)	χ^2^ = 0.95	0.330
Tumor location (Right/Left/Sigmoid/Rectum), *n* (%)	31 (29.2)/19 (17.9)/18 (17.0)/38 (35.8)	25 (23.1)/19 (17.6)/17 (15.7)/47 (43.5)	χ^2^ = 1.61	0.658
Pathologic TNM stage (I/II/III/IV), *n* (%)	22 (20.8)/34 (32.1)/35 (33.0)/15 (14.2)	29 (26.9)/28 (25.9)/35 (32.4)/16 (14.8)	χ^2^ = 1.56	0.670
Surgical approach (Laparoscopic/Open), *n* (%)	80 (75.5)/26 (24.5)	77 (71.3)/31 (28.7)	χ^2^ = 0.29	0.592
Procedure type (right hemicolectomy/left hemicolectomy/sigmoid colectomy/anterior resection/abdominoperineal resection), *n* (%)	31 (29.2)/22 (20.8)/21 (19.8)/24 (22.6)/8 (7.5)	35 (32.4)/16 (14.8)/15 (13.9)/28 (25.9)/14 (13.0)	χ^2^ = 4.12	0.391
Stoma (No/Temporary/Permanent), *n* (%)	78 (73.6)/20 (18.9)/8 (7.5)	81 (75.0)/21 (19.4)/6 (5.6)	χ^2^ = 0.35	0.840
Anesthesia (GA/GA+Epidural), *n* (%)	95 (89.6)/11 (10.4)	97 (89.8)/11 (10.2)	χ^2^ = 0.00	1.000
Operative duration, min	159.13 ± 45.33	165.60 ± 45.39	*t* = −1.04	0.298
Estimated blood loss, mL, median (IQR)	123 (84–188)	122 (78–188)	Z = −0.01	0.993
Perioperative transfusion, *n* (%)	8 (7.5)	16 (14.8)	χ^2^ = 2.15	0.142
Preoperative chemo/radiotherapy, *n* (%)	18 (17.0)	21 (19.4)	χ^2^ = 0.08	0.772
ASA physical status (I/II/III/IV), *n* (%)	8 (7.5)/53 (50.0)/39 (36.8)/6 (5.7)	13 (12.0)/61 (56.5)/27 (25.0)/7 (6.5)	χ^2^ = 3.99	0.262
Diabetes mellitus, *n* (%)	25 (23.6)	28 (25.9)	χ^2^ = 0.06	0.812
Anemia, *n* (%)	30 (28.3)	33 (30.6)	χ^2^ = 0.04	0.832
Chronic kidney disease, *n* (%)	4 (3.8)	10 (9.3)	χ^2^ = 1.81	0.178

ASA, American Society of Anesthesiologists; BMI, body mass index; CKD, chronic kidney disease; GA, general anesthesia; IQR, interquartile range; NRS-2002, Nutritional Risk Screening 2002; TNM, tumor-node-metastasis.

### 3.3 Primary outcome: post-operative nutritional risk status assessed by NRS-2002

The primary outcome was postoperative nutritional risk status assessed using NRS-2002. Preoperative NRS-2002 status did not differ significantly between the control group and the observation group. At the postoperative assessment time point, defined as POD7 or the last assessment before discharge, the observation group had a significantly lower postoperative NRS-2002 score and a significantly smaller change in NRS-2002 from baseline than the control group (both *P* < 0.001). The proportion of patients with postoperative nutritional risk, defined as NRS-2002 ≥3, was also significantly lower in the observation group (*P* = 0.001). Among patients with preoperative nutritional risk, the observation group showed a higher rate of transition to NRS-2002 < 3 and a lower rate of persistent postoperative nutritional risk than the control group (both *P* = 0.009). Among patients without preoperative nutritional risk, new-onset postoperative nutritional risk occurred less frequently in the observation group than in the control group (*P* = 0.003) (Table 2).

**Table 2:** Postoperative nutritional risk outcomes based on NRS-2002.

Outcome	Control (*n* = 106)	Observation group (*n* = 108)	Test statistic	*P* value
Preoperative NRS-2002, median (IQR)	2 (1–3)	2 (1–3)	Z = −0.96	0.325
Postoperative NRS-2002 (POD7/discharge), median (IQR)	3 (2–5)	2 (1–4)	Z = 4.02	<0.001
ΔNRS-2002 (postoperative – preoperative), median (IQR)	1 (0–2)	0 (−1–1)	Z = 5.82	<0.001
Preoperative NRS-2002 ≥ 3, *n* (%)	44 (41.5)	53 (49.1)	χ^2^ = 0.95	0.330
Postoperative NRS-2002 ≥ 3, *n* (%)	71 (67.0)	48 (44.4)	χ^2^ = 10.11	0.001
Nutritional risk improvement among baseline-risk patients (baseline ≥ 3 to postoperative < 3), *n*/*N* (%)	3/44 (6.8)	16/53 (30.2)	χ^2^ = 6.92	0.009
Nutritional risk persistence among baseline-risk patients (baseline ≥ 3 and postoperative ≥ 3), *n*/*N* (%)	41/44 (93.2)	37/53 (69.8)	χ^2^ = 6.92	0.009
New-onset nutritional risk among baseline non-risk patients (baseline < 3 to postoperative ≥ 3), *n*/*N* (%)	30/62 (48.4)	11/55 (20.0)	χ^2^ = 9.11	0.003

IQR, interquartile range; NRS-2002, Nutritional Risk Screening 2002; POD, postoperative day; ΔNRS-2002, postoperative minus preoperative NRS-2002 score.

### 3.4 Secondary outcomes: nutrition-related and clinical recovery and utilization

For secondary nutrition-related and nutrition-inflammation outcomes, the observation group had less discharge weight loss and smaller decreases in albumin, prealbumin, and total protein than the control group (all *P* < 0.05). These serum protein changes were interpreted as laboratory nutrition-inflammation markers rather than isolated nutritional markers in the acute postoperative period. The change in hemoglobin did not differ significantly between the two groups (*P* > 0.05). The observation group also had lower discharge CRP and a smaller increase in CRP from baseline than the control group (both *P* < 0.001). In addition, the prevalence of malnutrition at discharge and the distribution of discharge NRS-2002 risk categories differed between groups, with the observation group showing a lower proportion of nutrition-related deterioration at discharge (both *P* < 0.01). Regarding nutrition-support utilization, the overall distribution of the highest nutrition support level did not differ significantly between the control group and the observation group (*P* = 0.143). However, among patients receiving EN or PN, escalation from ONS to EN/PN occurred less frequently in the observation group (*P* = 0.044). For recovery- and utilization-related outcomes, the observation group had earlier first bowel movement, earlier tolerance of oral intake, and earlier tolerance of a soft/regular diet than the control group (all *P* < 0.05). Time to first flatus did not differ significantly between groups (*P* > 0.05). Postoperative length of stay was shorter in the observation group (*P* < 0.001), whereas total hospitalization cost and 30-day readmission were comparable between the two groups (both *P* > 0.05) (Table 3).

**Table 3:** Secondary outcomes: nutrition-related and nutrition-inflammation indices, nutrition support, and clinical recovery/utilization.

Outcome	Control (*n* = 106)	Observation group (*n* = 108)	Test statistic	*P* value
Nutrition-related and nutrition-inflammation outcomes				
ΔWeight (kg; discharge – preoperative)	−3.14 ± 1.53	−1.51 ± 1.44	*t* = −8.05	<0.001
ΔAlbumin (g/L; discharge – preoperative)	−6.24 ± 3.34	−4.48 ± 3.07	*t* = −4.00	<0.001
ΔPrealbumin (mg/L; discharge – preoperative)	−61.10 ± 43.96	−36.50 ± 37.05	*t* = −4.42	<0.001
ΔTotal protein (g/L; discharge – preoperative)	−4.44 ± 3.20	−3.75 ± 2.90	*t* = −2.08	0.039
ΔHemoglobin (g/L; discharge – preoperative)	−17.70 ± 10.62	−14.76 ± 10.03	*t* = −1.59	0.113
CRP at discharge (mg/L), median (IQR)	61.7 (45.7–77.9)	42.8 (33.1–61.7)	Z = 4.82	<0.001
ΔCRP (mg/L; discharge – preoperative), median (IQR)	55.5 (39.7–69.8)	36.3 (25.5–52.6)	Z = 4.80	<0.001
Malnutrition at discharge, *n* (%)	82 (77.4)	58 (53.7)	χ^2^ = 12.21	<0.001
Discharge NRS-2002 category (Low/Moderate/High), *n* (%)	35 (33.0)/44 (41.5)/27 (25.5)	60 (55.6)/31 (28.7)/17 (15.7)	χ^2^ = 11.09	0.004
Highest level of nutrition support (ONS only/EN/PN), *n* (%)	63 (59.4)/30 (28.3)/13 (12.3)	78 (72.2)/21 (19.4)/9 (8.3)	χ^2^ = 3.89	0.143
Escalation from ONS to EN/PN among EN/PN recipients, *n*/*N* (%)	30/43 (69.8)	13/30 (43.3)	χ^2^ = 4.07	0.044
Clinical recovery and utilization				
Time to first flatus (days), median (IQR)	2.3 (1.7–3.3)	2.4 (1.7–3.0)	Z = 0.57	0.571
Time to first bowel movement (days), median (IQR)	4.0 (3.0–4.9)	3.6 (2.8–4.3)	Z = 2.24	0.025
Time to tolerate oral intake (days), median (IQR)	1.7 (1.2–2.1)	1.4 (1.0–1.9)	Z = 2.30	0.021
Time to tolerate soft/regular diet (days), median (IQR)	4.3 (3.4–5.4)	3.6 (2.7–4.6)	Z = 3.07	0.002
Postoperative length of stay (days), median (IQR)	8.6 (6.5–11.3)	7.5 (6.0–8.8)	Z = 3.37	<0.001
Total hospitalization cost (kCNY), median (IQR)	59.8 (53.0–67.8)	59.8 (51.0–66.1)	Z = 0.81	0.416
30-day readmission, *n* (%)	10 (9.4)	10 (9.3)	χ^2^ = 0.00	1.000

Δ indicates discharge minus preoperative value unless otherwise specified. CRP, C-reactive protein; EN, enteral nutrition; IQR, interquartile range; kCNY, thousand Chinese yuan; NRS-2002, Nutritional Risk Screening 2002; ONS, oral nutritional supplements; PN, parenteral nutrition.

### 3.5 Post-operative complications, 30-day events, and nutrition-support–related safety outcomes

The overall rate of postoperative complications was lower in the observation group than in the control group (*P* = 0.025). For individual postoperative complications, no statistically significant between-group differences were observed for surgical site infection, anastomotic leak, or pneumonia (all *P* > 0.05), whereas postoperative ileus/obstruction showed a borderline difference between the control group and the observation group (*P* = 0.055). The overall distribution of Clavien–Dindo grades and the incidence of major complications did not differ significantly between groups (both *P* > 0.05). For 30-day outcomes, readmission, reoperation, and mortality did not differ significantly between the control group and the observation group (all *P* > 0.05). Regarding nutrition-support–related safety outcomes, the rate of any safety event was lower in the observation group than in the control group (*P* = 0.026). Gastrointestinal intolerance requiring adjustment of the nutrition plan and hyperglycemia requiring intervention were also less frequent in the observation group (both *P* < 0.05). No significant between-group differences were identified for nausea/vomiting, diarrhea, aspiration events, catheter-related complications, or electrolyte disorders requiring correction (all *P* > 0.05) (Table 4).

**Table 4:** Post-operative complications, 30-day events, and nutrition-support–related safety outcomes.

Outcome	Control (*n* = 106)	Observation group (*n* = 108)	OR (95% CI)†	Test statistic	*P* value
Postoperative complications					
Any complication (overall)	36 (34.0)	22 (20.4)	0.50 (0.27–0.93)	χ^2^	0.025
Surgical site infection (SSI)	18 (17.0)	11 (10.2)	0.56 (0.25–1.23)	χ^2^	0.146
Anastomotic leak	3 (2.8)	1 (0.9)	0.32 (0.03–3.13)	Fisher	0.367
Postoperative ileus/obstruction	14 (13.2)	6 (5.6)	0.39 (0.14–1.05)	χ^2^	0.055
Pneumonia	8 (7.5)	5 (4.6)	0.60 (0.19–1.89)	χ^2^	0.372
Clavien–Dindo grade (0/I–II/III–IV/V), n (%)	70 (66.0)/17 (16.0)/18 (17.0)/1 (0.9)	86 (79.6)/11 (10.2)/11 (10.2)/0 (0.0)	—	χ^2^ = 5.60	0.133
Major complication (Clavien–Dindo III–V)	19 (17.9)	11 (10.2)	0.52 (0.23–1.15)	χ^2^	0.103
30-day events					
Readmission within 30 days	10 (9.4)	10 (9.3)	0.98 (0.39–2.44)	χ^2^	0.965
Reoperation within 30 days	7 (6.6)	5 (4.6)	0.68 (0.21–2.22)	χ^2^	0.530
Death within 30 days	1 (0.9)	0 (0.0)	0.32 (0.01–8.33)	Fisher	0.495
Safety outcomes related to nutrition support					
Gastrointestinal intolerance requiring adjustment	26 (24.5)	12 (11.1)	0.38 (0.18–0.81)	χ^2^	0.010
Nausea/vomiting	25 (23.6)	15 (13.9)	0.52 (0.26–1.06)	χ^2^	0.069
Diarrhea	20 (18.9)	11 (10.2)	0.49 (0.22–1.08)	χ^2^	0.071
Aspiration event	1 (0.9)	1 (0.9)	0.98 (0.06–16.67)	Fisher	1.000
Catheter-related complication	14 (13.2)	8 (7.4)	0.53 (0.21–1.32)	χ^2^	0.162
Hyperglycemia requiring intervention	14 (13.2)	5 (4.6)	0.32 (0.11–0.92)	χ^2^	0.027
Electrolyte disorder requiring correction	8 (7.5)	8 (7.4)	1.11 (0.41–3.03)	χ^2^	0.832
Any nutrition-support–related safety event (overall)	47 (44.3)	32 (29.6)	0.53 (0.30–0.92)	χ^2^	0.026

†ORs are calculated as the odds of the event in the observation group relative to the control group (OR < 1 indicates lower odds in the observation group). CI, confidence interval; OR, odds ratio; SSI, surgical site infection.

In a *post hoc* multivariable logistic regression for any postoperative complication, the observation group remained associated with lower odds of complications after adjustment for age, BMI, preoperative NRS-2002, ASA class, surgical approach, and perioperative transfusion (Supplementary Table S3). Preoperative NRS-2002 was positively associated with complication risk, while ASA III–IV, open surgery, and perioperative transfusion showed directionally unfavorable but statistically non-significant associations.

### 3.6 Unadjusted and adjusted regression analyses for the primary outcome

To further evaluate the association between the nursing pathway and postoperative nutritional risk status, both unadjusted and adjusted regression models were constructed for the two prespecified primary outcomes: ΔNRS-2002 as a continuous outcome and postoperative nutritional risk, defined as NRS-2002 ≥3 at POD7/discharge, as a binary outcome. The adjusted models included demographic, lifestyle, tumor-related, surgical, and major comorbidity variables. In the unadjusted analyses, the observation group had a smaller increase in NRS-2002 and lower odds of postoperative nutritional risk than the control group. These associations remained statistically significant after multivariable adjustment, suggesting that the association between the nutrition-focused evidence-based nursing protocol and more favorable postoperative NRS-2002 outcomes was not fully explained by measured baseline or perioperative differences. Preoperative NRS-2002 was strongly associated with postoperative nutritional risk in the logistic regression model, indicating that patients with higher baseline nutritional risk were more likely to remain at nutritional risk postoperatively. In the linear model for ΔNRS-2002, preoperative NRS-2002 was inversely associated with score change, which may reflect the influence of baseline score level on the magnitude of postoperative change. Most other covariates were not consistently associated with both primary outcomes after adjustment, although isolated associations were observed in one model. Overall, the observation group variable remained consistently associated with more favorable postoperative NRS-2002 outcomes across both models (Table 5).

**Table 5:** Unadjusted and adjusted models for the primary outcome (ΔNRS-2002 and postoperative nutritional risk at discharge/POD7).

Variable	Unadjusted β (SE) for ΔNRS	95% CI (unadjusted β)	*P* value (unadjusted β)	Adjusted β (SE) for ΔNRS	95% CI (ΔNRS)	*P* value (ΔNRS)	Unadjusted OR for postop NRS≥3	95% CI (unadjusted OR)	*P* value (unadjusted OR)	Adjusted OR for postop NRS≥3	95% CI (OR)	*P* value (OR)
Observation group (vs. control)	−1.082 (0.160)	−1.397 to −0.767	< 0.001	−1.192 (0.188)	−1.560 to −0.823	< 0.001	0.39	0.23 to 0.69	< 0.001	0.17	0.08 to 0.36	< 0.001
Preoperative NRS-2002 (per 1 point)	−0.312 (0.046)	−0.403 to −0.221	< 0.001	−0.225 (0.053)	−0.329 to −0.122	< 0.001	4.21	2.95 to 6.01	< 0.001	3.88	2.56 to 5.87	< 0.001
Age (per 1 year)	−0.006 (0.009)	−0.024 to 0.012	0.509	−0.004 (0.010)	−0.024 to 0.017	0.733	1.01	0.98 to 1.05	0.442	1.01	0.97 to 1.05	0.740
Male sex (vs. female)	−0.214 (0.167)	−0.543 to 0.114	0.201	−0.270 (0.183)	−0.629 to 0.090	0.142	0.84	0.49 to 1.45	0.531	0.81	0.39 to 1.67	0.567
Body mass index (per 1 kg/m^2^)	−0.021 (0.029)	−0.078 to 0.036	0.470	−0.014 (0.032)	−0.077 to 0.048	0.655	1.03	0.93 to 1.14	0.6	1.05	0.93 to 1.18	0.423
Former smoker (vs. never)	−0.156 (0.228)	−0.604 to 0.291	0.493	−0.184 (0.254)	−0.682 to 0.314	0.468	0.62	0.24 to 1.59	0.318	0.55	0.19 to 1.56	0.258
Current smoker (vs. never)	−0.221 (0.209)	−0.632 to 0.190	0.292	−0.260 (0.229)	−0.708 to 0.188	0.255	0.74	0.31 to 1.75	0.49	0.68	0.24 to 1.89	0.457
Alcohol use (yes vs. no)	−0.168 (0.183)	−0.528 to 0.191	0.360	−0.197 (0.201)	−0.591 to 0.198	0.328	0.58	0.32 to 1.04	0.068	0.46	0.22 to 0.99	0.047
Rectal tumor (vs. colon)	0.196 (0.181)	−0.160 to 0.552	0.279	0.239 (0.199)	−0.151 to 0.629	0.230	0.88	0.50 to 1.54	0.655	0.78	0.36 to 1.71	0.538
TNM stage II (vs. stage I)	−0.408 (0.215)	−0.831 to 0.015	0.059	−0.500 (0.238)	−0.967 to −0.034	0.035	0.53	0.23 to 1.22	0.136	0.44	0.17 to 1.19	0.105
TNM stage III–IV (vs. stage I)	−0.147 (0.199)	−0.539 to 0.245	0.461	−0.192 (0.219)	−0.622 to 0.237	0.381	0.7	0.32 to 1.53	0.372	0.55	0.24 to 1.29	0.169
Open surgery (vs. laparoscopic)	0.122 (0.194)	−0.260 to 0.504	0.531	0.099 (0.218)	−0.329 to 0.527	0.650	1.42	0.72 to 2.80	0.313	1.36	0.57 to 3.26	0.486
Stoma (any vs. none)	0.081 (0.218)	−0.348 to 0.510	0.709	0.033 (0.245)	−0.447 to 0.513	0.892	1.19	0.63 to 2.24	0.598	1.04	0.49 to 2.22	0.912
Operative duration (per 30 min)	0.028 (0.053)	−0.076 to 0.132	0.597	0.016 (0.059)	−0.100 to 0.132	0.786	1	0.82 to 1.22	0.987	0.97	0.77 to 1.22	0.794
Estimated blood loss (per 100 mL)	−0.086 (0.096)	−0.275 to 0.103	0.370	−0.107 (0.107)	−0.316 to 0.102	0.316	1.08	0.71 to 1.63	0.73	1.01	0.63 to 1.65	0.947
Perioperative transfusion (yes vs. no)	−0.043 (0.214)	−0.464 to 0.378	0.841	−0.064 (0.233)	−0.520 to 0.392	0.782	1.39	0.62 to 3.11	0.421	1.30	0.52 to 3.27	0.583
Preoperative chemotherapy/radiotherapy (yes vs. no)	0.139 (0.238)	−0.329 to 0.606	0.559	0.175 (0.259)	−0.333 to 0.682	0.500	1.29	0.59 to 2.84	0.526	1.21	0.46 to 3.24	0.703
ASA class II (vs. I)	−0.012 (0.307)	−0.616 to 0.592	0.969	−0.039 (0.335)	−0.696 to 0.618	0.907	1.02	0.43 to 2.41	0.964	0.96	0.36 to 2.55	0.933
ASA class III–IV (vs. I)	−0.221 (0.304)	−0.819 to 0.377	0.467	−0.263 (0.332)	−0.914 to 0.388	0.429	1.16	0.49 to 2.74	0.737	1.07	0.42 to 2.72	0.892
Diabetes mellitus (yes vs. no)	−0.247 (0.187)	−0.615 to 0.121	0.187	−0.359 (0.208)	−0.766 to 0.048	0.084	0.58	0.27 to 1.24	0.159	0.37	0.15 to 0.90	0.028
Anemia (yes vs. no)	−0.176 (0.185)	−0.541 to 0.188	0.341	−0.223 (0.203)	−0.622 to 0.176	0.272	0.9	0.47 to 1.71	0.739	0.84	0.41 to 1.72	0.640
Chronic kidney disease (yes vs. no)	−0.295 (0.353)	−0.989 to 0.399	0.411	−0.378 (0.392)	−1.147 to 0.391	0.335	0.63	0.17 to 2.26	0.483	0.49	0.12 to 2.00	0.318

ASA, American Society of Anesthesiologists; CI, confidence interval; NRS-2002, Nutritional Risk Screening 2002; OR, odds ratio; POD, postoperative day; SE, standard error; TNM, tumor-node-metastasis; ΔNRS-2002, postoperative minus preoperative NRS-2002 score.

Model diagnostics further supported the adequacy of the primary multivariable analyses. The logistic regression model showed good discrimination (C-statistic/AUC 0.814, 95% CI 0.756–0.872) and acceptable calibration, with a calibration intercept of −0.03, a calibration slope of 0.96, and a non-significant Hosmer–Lemeshow test (χ^2^ = 6.48, *P* = 0.593); the Brier score was 0.176. The calibration plot also showed close agreement between observed and predicted risks (Supplementary Table S4; Supplementary Figure S5). The linear regression model showed acceptable performance (adjusted R^2^ = 0.342), acceptable residual normality on the Q–Q plot, no major heteroscedastic pattern, and no influential outliers of concern (maximum Cook's distance = 0.09). In addition, multicollinearity appeared minimal, with VIF values ranging from 1.06 to 1.94 (mean VIF 1.36) and a minimum tolerance of 0.515, indicating no evidence of problematic collinearity among the included covariates (Supplementary Tables S4, S6).

### 3.7 Subgroup analyses of the primary outcome

Subgroup analyses were performed to examine whether the association between the nutrition-focused evidence-based nursing protocol and the primary outcome was consistent across clinically relevant strata, including tumor site, surgical approach, stoma status, and preoperative nutritional risk. Logistic regression was used for postoperative nutritional risk at discharge/POD7 (NRS-2002 ≥3), and linear regression was used for ΔNRS-2002. Each model included a group-by-subgroup interaction term and was adjusted for prespecified baseline covariates as appropriate. Across all subgroups, the direction of association was consistent between the control group and the observation group for both postoperative nutritional risk and ΔNRS-2002. No statistically significant interaction was identified for tumor site, surgical approach, stoma status, or preoperative nutritional risk (all *P* for interaction > 0.05) (Table 6, Supplementary Figure S7).

**Table 6:** Subgroup analyses of the primary outcome by tumor site, surgical approach, stoma status, and preoperative nutritional risk.

Subgroup	Level	Postoperative NRS-2002 ≥3 (Control)	Postoperative NRS-2002 ≥3 (Observation)	Adjusted OR (Observation vs. Control)	*P* for interaction (OR)	Adjusted β (Observation–Control) for ΔNRS	*P* for interaction (β)
Tumor site	Colon	47/68 (69.1)	26/61 (42.6)	0.15 (0.06–0.39)	—	−1.27 (−1.73–−0.81)	—
Tumor site	Rectum	24/38 (63.2)	22/47 (46.8)	0.26 (0.08–0.84)	0.470	−1.17 (−1.72–−0.62)	0.786
Surgical approach	Laparoscopic	51/80 (63.7)	33/77 (42.9)	0.17 (0.07–0.41)	—	−1.23 (−1.64–−0.82)	—
Surgical approach	Open	20/26 (76.9)	15/31 (48.4)	0.23 (0.05–0.98)	0.722	−1.23 (−1.91–−0.54)	0.996
Stoma status	No stoma	52/78 (66.7)	35/81 (43.2)	0.14 (0.06–0.35)	—	−1.37 (−1.78–−0.96)	—
Stoma status	Stoma	19/28 (67.9)	13/27 (48.1)	0.39 (0.09–1.69)	0.245	−0.83 (−1.53–−0.14)	0.198
Preoperative nutritional risk	Low risk (NRS-2002 < 3)	30/62 (48.4)	11/55 (20.0)	0.25 (0.10–0.59)	—	−1.26 (−1.75–−0.78)	—
Preoperative nutritional risk	High risk (NRS-2002 ≥ 3)	41/44 (93.2)	37/53 (69.8)	0.15 (0.04–0.59)	0.566	−1.18 (−1.71–−0.65)	0.826

ΔNRS-2002 = (discharge/POD7 NRS-2002 – preoperative NRS-2002). OR < 1 and β < 0 favor the observation group. Interaction *P* values are shown on the second row of each subgroup. CI, confidence interval; NRS-2002, Nutritional Risk Screening 2002; OR, odds ratio; POD, postoperative day; ΔNRS-2002, postoperative minus preoperative NRS-2002 score.

### 3.8 Sensitivity analyses

Sensitivity analyses were conducted to assess the robustness of the primary findings across alternative model specifications. The primary binary endpoint was postoperative nutritional risk at discharge/POD7, defined as NRS-2002 ≥ 3, and the continuous endpoint was ΔNRS-2002. All regression models used heteroskedasticity-robust standard errors (HC3). Across the full-adjusted model, parsimonious adjustment model, model excluding perioperative intensity variables, and model excluding baseline NRS-2002 from the adjustment set, the association between the nursing protocol and both primary outcomes remained directionally consistent and statistically significant. When baseline NRS-2002 was removed from the adjustment set, the association for the binary outcome was attenuated, whereas the result for ΔNRS-2002 remained similar; statistical significance was retained for both outcomes (Table 7).

**Table 7:** Sensitivity analyses for the primary outcome using alternative model specifications.

Sensitivity analysis scenario	Adjusted OR for postop NRS-2002 ≥3 (Observation vs. Control)	*P* value	Adjusted β for ΔNRS-2002 (Observation – Control)	*P* value
Full-adjusted models (primary)	0.16 (95% CI 0.07–0.33)	<0.001	−1.153 (95% CI −1.544 to −0.762)	<0.001
Parsimonious adjustment set	0.18 (95% CI 0.09–0.37)	<0.001	−1.203 (95% CI −1.555 to −0.850)	<0.001
Excluding perioperative intensity variables	0.16 (95% CI 0.08–0.34)	<0.001	−1.144 (95% CI −1.520 to −0.767)	<0.001
Excluding baseline NRS-2002 from adjustment	0.38 (95% CI 0.20–0.72)	0.003	−1.189 (95% CI −1.595 to −0.784)	<0.001

ASA, American Society of Anesthesiologists; CI, confidence interval; HC3, heteroskedasticity-consistent type 3; NRS-2002, Nutritional Risk Screening 2002; OR, odds ratio; POD, postoperative day; ΔNRS-2002, postoperative minus preoperative NRS-2002 score.

An additional sensitivity analysis restricted to patients assessed exactly on POD7 was performed in response to the concern regarding heterogeneity in postoperative assessment timing. In this POD7-only subset, postoperative nutritional risk remained lower in the observation group than in the control group (44.3% vs. 66.7%, *P* = 0.013), and ΔNRS-2002 remained smaller in the observation group (*P* < 0.001) (Supplementary Table S8). These findings were directionally consistent with the primary analysis, suggesting that variability in assessment timing did not materially affect the main results.

### 3.9 *Post-hoc* power analysis

A *post hoc* power analysis was performed for the two prespecified primary outcomes. For the continuous primary outcome, ΔNRS-2002, the observed between-group difference corresponded to an approximate standardized effect size of Cohen's *d* = 0.675, yielding an estimated statistical power of 99.9% (>80%) at a two-sided α level of 0.05. For the binary primary outcome, postoperative nutritional risk defined as NRS-2002 ≥ 3 at POD7/discharge, the observed event rates were 67.0% in the control group and 44.4% in the observation group; this corresponded to an approximate effect size of Cohen's *h* = 0.458 and an estimated statistical power of 91.8% (>80%) at a two-sided α level of 0.05. These findings suggest that the study had adequate statistical power for detecting the observed between-group differences in both primary outcomes.

### 3.10 Missing secondary outcome data and interpretation framework

Missingness for secondary outcomes was limited overall. Most in-hospital secondary and safety endpoints were complete, whereas small amounts of missing data were observed mainly for selected discharge laboratory indices, including albumin, pre-albumin, total protein, and CRP-related measures. All secondary and safety outcomes were therefore analyzed using an available-case approach without imputation, and the analyzable denominators for each endpoint are summarized in Supplementary Table S9. In addition, the analytic hierarchy and interpretation framework are provided in Supplementary Table S10, which clarifies that the two primary outcomes were prespecified as the main inferential endpoints, whereas secondary, safety, subgroup, and sensitivity analyses were interpreted as exploratory or supportive.

## 4 Discussion

In this retrospective study of patients undergoing colorectal cancer surgery, a nutrition-focused evidence-based nursing protocol was associated with more favorable postoperative nutritional risk status assessed by NRS-2002, together with more favorable short-term nutrition-related trajectories and selected recovery outcomes. Compared with the control group, the observation group had lower postoperative NRS-2002 scores, a smaller increase in NRS-2002 from baseline, and a lower prevalence of postoperative nutritional risk, with concordant secondary findings including less postoperative weight loss, smaller decreases in albumin, pre-albumin, and total protein, lower discharge CRP levels and smaller CRP increases, earlier tolerance of oral intake and soft/regular diet, and a shorter postoperative length of stay. For the primary outcomes, these associations remained significant after adjustment for pre-specified demographic, tumor-related, and perioperative covariates and were generally consistent across clinically relevant strata, with no statistically significant interaction detected. The present study is clinically relevant because it evaluated a pragmatic, nurse-led perioperative nutritional care pathway within a stable ERAS-based colorectal perioperative framework, using dynamic postoperative nutritional risk as a pre-specified primary outcome rather than relying solely on isolated laboratory markers or single nutrition-support measures. In the context of contemporary perioperative optimization, postoperative nutritional risk reduction remains a clinically meaningful objective even in the absence of a measurable difference in hospitalization costs, because preservation of nutritional status is closely linked to recovery quality, treatment tolerance, and overall surgical resilience (17). Importantly, the protocol was embedded in routine clinical practice and incorporated early nutritional risk screening, individualized intake targets, protocolized escalation from oral strategies to EN or PN when needed, and discharge-transition support, thereby highlighting the central role of nursing in operationalizing nutritional screening, reinforcing protocol adherence, and maintaining continuity of perioperative nutritional care (18, 19).

A key implication of the primary outcome is that the protocol was associated not only with a lower overall prevalence of postoperative nutritional risk, but also with different risk-transition patterns according to baseline nutritional status. Among patients without preoperative nutritional risk, new-onset postoperative nutritional risk was less frequent in the observation group, whereas among patients with preoperative nutritional risk, transition to postoperative NRS-2002 < 3 was more common. This pattern suggests that structured perioperative nutritional surveillance may be clinically relevant for both early identification of emerging risk and closer follow-up of patients with pre-existing vulnerability (20, 21). Importantly, NRS-2002 should be interpreted as a pragmatic nutritional risk-screening outcome rather than a direct measure of body composition or isolated nutritional depletion. The observed differences in NRS-2002 were nevertheless supported by concordant secondary findings, including less weight loss and smaller declines in albumin, prealbumin, and total protein. Although these biochemical markers are influenced by surgical stress, inflammation, and perioperative fluid dynamics, their directionally consistent pattern strengthens the overall interpretation that the observation group had a more favorable short-term nutrition-related trajectory (22, 23). In addition, preoperative NRS-2002 remained strongly associated with postoperative nutritional risk in the logistic regression model, underscoring the importance of baseline vulnerability in postoperative nutritional-risk stratification.

The pattern of secondary and safety outcomes further suggests that the clinical relevance of the protocol may be most apparent in process-sensitive aspects of perioperative care. The observation group had earlier bowel movement, earlier tolerance of oral intake and soft/regular diet, and shorter postoperative hospitalization, whereas readmission and total hospitalization costs were similar between groups. These findings are compatible with the role of structured nursing reassessment in supporting intake monitoring, symptom-responsive dietary progression, and timely review when intake remained inadequate. The overall distribution of the highest nutrition support level did not differ significantly between groups; however, among patients receiving EN or PN, escalation from ONS to EN/PN occurred less frequently in the observation group. This may reflect earlier recognition and management of inadequate intake before later intensification became necessary, although this interpretation remains exploratory (24). Similarly, the lower overall postoperative complication rate and lower rate of nutrition-support–related safety events, particularly gastrointestinal intolerance requiring adjustment and hyperglycemia requiring intervention, are compatible with more individualized and better-tolerated nutritional management under closer nursing surveillance. The lower discharge CRP level should be interpreted cautiously, as it may reflect a lower overall postoperative inflammatory burden related to fewer complications, better metabolic tolerance, or faster recovery rather than a direct anti-inflammatory effect of the protocol itself. Several endpoints remained unchanged, including hemoglobin change, major complications, readmission, and total costs (25). These neutral findings argue against an overly broad interpretation and indicate that the observed associations were strongest for early nutritional recovery, selected functional recovery milestones, and process-sensitive safety outcomes rather than for all downstream clinical or resource-use indicators (26). Within ERAS-oriented colorectal care, these findings support the practical value of embedding structured screening, reassessment, and follow-up into routine nursing workflows to promote adherence to perioperative nutritional care pathways (27, 28).

Recent evidence supports the importance of perioperative nutrition and structured care pathways in colorectal surgery, while also showing that effects may differ across endpoints. Su et al. reported in a systematic review and meta-analysis that preoperative oral nutritional supplements (ONS) were associated with a lower overall risk of postoperative complications in colorectal cancer surgery, whereas effects on specific complications, such as wound infection and anastomotic leakage, were less consistent; this pattern is broadly consistent with our finding of fewer overall complications without uniform significance across individual complication categories (29). Müller et al. (30) showed that motivational interviewing within an Enhanced Recovery After Surgery (ERAS) pathway improved compliance with perioperative nutrition goals, suggesting that structured, patient-centered counseling may support nutrition-related care implementation. Similarly, Pi et al. (31) reported that individualized nutrition education based on goal attainment theory was associated with better ONS adherence and higher postoperative prealbumin, which is consistent with the smaller prealbumin decline and more favorable NRS-2002 trajectories observed in the present study. Zhang et al. (32) further reported that ERAS combined with multidisciplinary collaboration was associated with faster recovery milestones and lower inflammatory markers, providing complementary evidence for bundled perioperative strategies. Collectively, these studies support the clinical plausibility of protocolized, nursing-led nutritional care, while effects on individual complications, long-term nutritional outcomes, and economic endpoints require confirmation in larger prospective multicenter studies.

Several limitations should be acknowledged. First, this was a retrospective, single-center, sequential before-and-after study using historical controls. Although baseline characteristics were broadly comparable and the institutional ERAS-based perioperative framework remained unchanged, residual confounding and secular trends cannot be fully excluded, particularly for utilization-related endpoints such as length of stay. Second, postoperative NRS-2002 was used as a pragmatic indicator of postoperative nutritional risk status rather than a validated responsiveness measure for short-term postoperative nutritional change. Postoperative NRS-2002 changes may partly reflect surgical stress and perioperative disease burden rather than isolated nutritional deterioration. Because item-level NRS-2002 data were incomplete, recalculation excluding the disease severity component was not feasible. Although the POD7-restricted sensitivity analysis supported the robustness of the main findings, variation in assessment timing may still have introduced heterogeneity. Third, discharge weight may have been affected by perioperative fluid shifts because edema, fluid retention, and fluid balance were not directly measured. Similarly, albumin and pre-albumin were interpreted as laboratory nutrition-inflammation markers rather than isolated nutritional markers, given their susceptibility to postoperative inflammation and fluid dynamics. Future studies should incorporate more granular and longitudinal nutritional assessment tools, including bioelectrical impedance analysis, computed tomography-derived muscle indices, handgrip strength, validated nutritional assessment scales, and fluid-balance evaluation. Fourth, intervention fidelity was not prospectively quantified using standardized metrics, limiting assessment of adherence to individual protocol components. Fifth, although statistical power was adequate for the pre-specified primary outcomes, uncommon complications, reoperation, mortality, and subgroup effects were likely underpowered and should be considered exploratory. Finally, follow-up was limited to in-hospital and 30-day outcomes, and the mechanisms underlying lower CRP remain uncertain. Larger prospective multicenter studies with contemporaneous controls, standardized complication adjudication, longer follow-up, longitudinal inflammatory and metabolic markers, quality-of-life assessment, adjuvant therapy tolerance evaluation, and formal cost-effectiveness analyses are warranted.

## 5 Conclusion

In patients undergoing colorectal cancer surgery, nutrition-focused evidence-based nursing was associated with more favorable postoperative nutritional risk status as assessed by NRS-2002. Compared with routine care, the observation group had lower POD7/discharge NRS-2002 scores, smaller ΔNRS-2002, and a lower prevalence of postoperative nutritional risk, with directionally consistent findings across baseline risk strata. Secondary findings were consistent with less nutritional deterioration, earlier feeding recovery, fewer overall complications, and fewer nutrition-support–related adverse events.

## Data Availability

The raw data supporting the conclusions of this article will be made available by the authors, without undue reservation.
