# Supplementary material for: Association between nutrition-focused evidence-based nursing and post-operative nutritional status in patients undergoing colorectal cancer surgery
Source: Front Nutr. 2026 Aug 7;13:1784385. doi: 10.3389/fnut.2026.1784385 (PMC13493245; doi:10.3389/fnut.2026.1784385)
Supplement: Supplementary file 1 [file Table_1.DOCX]

Supplementary Material 1. Structured Reassessment and Escalation Algorithm for the Nutrition-Focused Nursing Intervention

1. Daily Assessment Components

The following items were assessed by ward nurses during routine perioperative care:

Nutrition intake: estimated daily oral intake, including meal consumption, oral nutritional supplement (ONS) intake, and approximate achievement of individualized energy/protein targets.

Gastrointestinal tolerance: nausea, vomiting, diarrhea, abdominal distension, bowel sounds, passage of flatus, and stool passage.

Weight monitoring: daily or every-other-day monitoring when clinically feasible, depending on patient stability.

Laboratory indices: review of available nutrition- and inflammation-related laboratory results, including serum albumin, prealbumin, total protein, and C-reactive protein (CRP), when tested as part of routine care.

Symptoms affecting intake: pain, nausea, anorexia, abdominal discomfort, and stoma output when applicable.

2. Documentation Tools

Intake and tolerance were documented using standardized nursing record forms integrated into the electronic medical record (EMR). These forms included target versus achieved intake, type of nutrition support used (oral diet, ONS, enteral nutrition [EN], or parenteral nutrition [PN]), gastrointestinal tolerance, and nutrition-related symptoms. An escalation checklist was used to guide nutrition intervention adjustment when intake targets were not met.

3. Criteria for Nutritional Insufficiency or Reassessment

Nutritional insufficiency or need for reassessment was considered when one or more of the following occurred:

Total oral intake remained <60% of the individualized daily energy/protein target for 48–72 hours.

Prescribed ONS intake was <2 servings/day or <60% of the prescribed amount for 2 consecutive days.

Evidence of nutrition-related deterioration, such as ≥5% weight loss or serum albumin <35 g/L, was identified during hospitalization. These findings triggered reassessment and clinical review rather than automatic escalation.

4. Escalation Algorithm

| Step | Trigger | Action | Responsible personnel | Reassessment |
| --- | --- | --- | --- | --- |
| 1 | Oral intake <60% of target for 48–72 h | Initiate or intensify high-protein ONS, typically 200–250 mL per serving, 2–3 times daily | Ward nurse identifies trigger; dietitian/physician confirms plan when needed | Daily intake record and symptom review |
| 2 | Total oral intake including ONS remains <60% of target after 48–72 h | Consider EN if gastrointestinal function is adequate; consider PN if EN is contraindicated or poorly tolerated | Ward nurse, dietitian, and attending physician | Daily intake and tolerance assessment; laboratory review every 24–48 h when available |
| 3 | Combined oral/enteral intake remains <60% of target after 72 h, or intolerance/complications occur | Reassess for complications, optimize EN/PN prescription, and consult the nutrition support team | Nutrition support team and attending physician | Daily reassessment of intake adequacy and tolerance |

5. Verification and Oversight

Initial assessments and daily intake records were completed by ward nurses. Escalation decisions were co-verified by a senior nurse, dietitian, or attending physician according to clinical condition. Protocol implementation was reviewed periodically by the nutrition support team to support consistency of care.

6. Implementation Notes

Energy and protein targets were generally set at 25–30 kcal/kg/day and 1.2–1.5 g/kg/day protein, respectively. ONS refers to high-protein oral nutritional supplements, EN refers to enteral nutrition, and PN refers to parenteral nutrition. Escalation decisions were guided by intake adequacy, gastrointestinal tolerance, and the patient’s overall postoperative clinical condition.

7. Statistical Considerations

Nutrition-support utilization variables, including ONS, EN, PN, and escalation from ONS to EN/PN, were extracted from routine clinical and nursing records when available and analyzed as nutrition-support–related outcomes. Protocol adherence was reviewed descriptively to support interpretation of intervention implementation.

Supplementary Table S2. Distribution of postoperative NRS-2002 assessment days by study group

| Assessment day | Control (n = 106) | Observation (n = 108) |
| --- | --- | --- |
| POD5 | 5 (4.7%) | 6 (5.6%) |
| POD6 | 10 (9.4%) | 12 (11.1%) |
| POD7 | 60 (56.6%) | 61 (56.5%) |
| POD8 | 24 (22.6%) | 23 (21.3%) |
| ≥ POD9 | 7 (6.6%) | 6 (5.6%) |
| Assessed before POD7 due to early discharge | 15 (14.2%) | 18 (16.7%) |
| Median assessment day (IQR) | 7 (7–8) | 7 (7–8) |

Abbreviations: NRS-2002, Nutritional Risk Screening 2002; POD, postoperative day; IQR, interquartile range. Early discharge was defined as discharge before POD7. Percentages are calculated within each study group.

Supplementary Table S3. Post hoc multivariable logistic regression for overall postoperative complications

| Variable | Unadjusted OR | 95% CI | P value | Adjusted OR | 95% CI | P value |
| --- | --- | --- | --- | --- | --- | --- |
| Observation group (vs control) | 0.5 | 0.27 to 0.93 | 0.025 | 0.45 | 0.22 to 0.91 | 0.026 |
| Age (per 10 years) | 1.12 | 0.89 to 1.40 | 0.34 | 1.18 | 0.90 to 1.55 | 0.232 |
| BMI (per 1 kg/m²) | 1.03 | 0.96 to 1.10 | 0.392 | 1.04 | 0.95 to 1.13 | 0.401 |
| Preoperative NRS-2002 (per 1 point) | 1.31 | 1.05 to 1.63 | 0.016 | 1.29 | 1.01 to 1.64 | 0.039 |
| ASA class III–IV (vs I–II) | 1.79 | 0.96 to 3.34 | 0.068 | 1.88 | 0.95 to 3.74 | 0.071 |
| Open surgery (vs laparoscopic) | 1.63 | 0.84 to 3.15 | 0.146 | 1.76 | 0.85 to 3.64 | 0.129 |
| Perioperative transfusion (yes vs no) | 2.06 | 0.92 to 4.60 | 0.078 | 2.11 | 0.92 to 4.84 | 0.077 |

Abbreviations: ASA, American Society of Anesthesiologists; BMI, body mass index; CI, confidence interval; NRS-2002, Nutritional Risk Screening 2002; OR, odds ratio.

Supplementary Table S4. Model diagnostics and multicollinearity assessment for the primary multivariable models

| Diagnostic item | Logistic model for postop NRS-2002 ≥3 | Linear model for ΔNRS-2002 |
| --- | --- | --- |
| Outcome type | Binary | Continuous |
| No. of patients | 214 | 214 |
| No. of outcome events | 119 | — |
| C-statistic / AUC (95% CI) | 0.814 (0.756–0.872) | — |
| Calibration intercept | -0.03 | — |
| Calibration slope | 0.96 | — |
| Hosmer–Lemeshow χ² | 6.48 | — |
| Hosmer–Lemeshow P value | 0.593 | — |
| Brier score | 0.176 | — |
| Adjusted R² | — | 0.342 |
| Residual normality | — | Acceptable on Q–Q plot |
| Homoscedasticity | — | No major heteroscedastic pattern observed |
| Maximum Cook’s distance | — | 0.09 |
| VIF range | 1.06–1.94 | 1.06–1.94 |
| Mean VIF | 1.36 | 1.36 |
| Minimum tolerance | 0.515 | 0.515 |

Abbreviations: AUC, area under the receiver operating characteristic curve; CI, confidence interval; ΔNRS-2002, postoperative minus preoperative Nutritional Risk Screening 2002 score; NRS-2002, Nutritional Risk Screening 2002; Q–Q, quantile–quantile; VIF, variance inflation factor.
Supplementary Figure S5. Calibration plot for the multivariable logistic regression model

Supplementary Table S6. Variance inflation factor (VIF) assessment for covariates included in the multivariable models

| Predictor | VIF | Tolerance |
| --- | --- | --- |
| Study group (observation vs control) | 1.08 | 0.926 |
| Age | 1.17 | 0.855 |
| Sex | 1.09 | 0.917 |
| Body mass index | 1.21 | 0.826 |
| Smoking status | 1.34 | 0.746 |
| Alcohol use | 1.29 | 0.775 |
| Preoperative NRS-2002 | 1.42 | 0.704 |
| Tumor site | 1.18 | 0.847 |
| TNM stage | 1.52 | 0.658 |
| Surgical approach | 1.27 | 0.787 |
| Stoma status | 1.31 | 0.763 |
| Operative duration | 1.39 | 0.719 |
| Estimated blood loss | 1.44 | 0.694 |
| Perioperative transfusion | 1.26 | 0.794 |
| Preoperative chemo/radiotherapy | 1.22 | 0.82 |
| ASA class | 1.94 | 0.515 |
| Diabetes mellitus | 1.14 | 0.877 |
| Anemia | 1.16 | 0.862 |
| Chronic kidney disease | 1.06 | 0.943 |

Abbreviations: ASA, American Society of Anesthesiologists; NRS-2002, Nutritional Risk Screening 2002; TNM, tumor–node–metastasis; VIF, variance inflation factor.

Supplementary Figure S7. Forest plot of subgroup analyses for postoperative nutritional risk (NRS-2002 ≥3 at POD7/discharge).

Notes: Odds ratios <1 favor the observation group. The values shown in the “P for interaction” column are interaction P values testing whether the association between the nursing pathway and postoperative nutritional risk differed significantly across levels of each subgroup variable. Because one interaction P value corresponds to the comparison between subgroup levels within the same variable, it is displayed only once for each subgroup factor rather than on every row.

Supplementary Table S8. Sensitivity analysis restricted to patients assessed exactly on POD7

| Outcome | Control (n = 60) | Observation (n = 61) | Test statistic | P value |
| --- | --- | --- | --- | --- |
| Postoperative NRS-2002 ≥3, n (%) | 40 (66.7) | 27 (44.3) | χ² = 6.15 | 0.013 |
| ΔNRS-2002, median (IQR) | 1 (0–2) | 0 (−1–1) | Z = 4.21 | <0.001 |

Abbreviations: IQR, interquartile range; NRS-2002, Nutritional Risk Screening 2002; POD, postoperative day; ΔNRS-2002, postoperative minus preoperative NRS-2002 score.

Supplementary Table S9. Availability of secondary outcome data and handling of missingness

| Outcome | Analysis approach | Analyzable n / total n | Missing n (%) |
| --- | --- | --- | --- |
| ΔWeight (discharge − preoperative) | Available-case | 214/214 | 0 (0.0) |
| ΔAlbumin (discharge − preoperative) | Available-case | 210/214 | 4 (1.9) |
| ΔPrealbumin (discharge − preoperative) | Available-case | 206/214 | 8 (3.7) |
| ΔTotal protein (discharge − preoperative) | Available-case | 210/214 | 4 (1.9) |
| ΔHemoglobin (discharge − preoperative) | Available-case | 214/214 | 0 (0.0) |
| CRP at discharge | Available-case | 208/214 | 6 (2.8) |
| ΔCRP (discharge − preoperative) | Available-case | 208/214 | 6 (2.8) |
| Malnutrition at discharge | Available-case | 214/214 | 0 (0.0) |
| Highest level of nutrition support | Available-case | 214/214 | 0 (0.0) |
| Escalation from ONS to EN/PN among EN/PN recipients | Available-case | 73/73 | 0 (0.0) |
| Time to first flatus | Available-case | 214/214 | 0 (0.0) |
| Time to first bowel movement | Available-case | 214/214 | 0 (0.0) |
| Time to tolerate oral intake | Available-case | 214/214 | 0 (0.0) |
| Time to tolerate soft/regular diet | Available-case | 214/214 | 0 (0.0) |
| Postoperative length of stay | Available-case | 214/214 | 0 (0.0) |
| Total hospitalization cost | Available-case | 214/214 | 0 (0.0) |
| 30-day readmission | Available-case | 214/214 | 0 (0.0) |
| Reoperation within 30 days | Available-case | 214/214 | 0 (0.0) |
| Death within 30 days | Available-case | 214/214 | 0 (0.0) |

Abbreviations: CRP, C-reactive protein; EN, enteral nutrition; ONS, oral nutritional supplements; PN, parenteral nutrition.

Supplementary Table S10. Outcome hierarchy and interpretation framework

| Outcome category | Outcomes | Multiplicity handling | Interpretation |
| --- | --- | --- | --- |
| Primary outcomes | ΔNRS-2002; postoperative nutritional risk (NRS-2002 ≥3 at POD7/discharge) | No multiplicity adjustment across non-primary analyses; primary outcomes prespecified | Main inferential outcomes |
| Secondary outcomes | Nutrition-related and clinical recovery outcomes in Table 3 | No formal multiplicity adjustment | Exploratory / supportive |
| Safety outcomes | Nutrition-support–related adverse events in Table 4 | No formal multiplicity adjustment | Exploratory / supportive |
| Subgroup analyses | Tumor site, surgical approach, stoma status, baseline nutritional risk | No formal multiplicity adjustment | Exploratory / hypothesis-generating |
| Sensitivity analyses | Alternative specifications in Table 7 | Not part of multiplicity-controlled family | Robustness assessment |

Abbreviations: NRS-2002, Nutritional Risk Screening 2002; POD, postoperative day; ΔNRS-2002, postoperative minus preoperative NRS-2002 score.
